# Within-subject, double-blind, randomized, placebo-controlled evaluation of combining the cannabinoid dronabinol and the opioid hydromorphone in adults with chronic pain

**DOI:** 10.1038/s41386-023-01597-1

**Published:** 2023-05-18

**Authors:** Claudia M. Campbell, Chung Jung Mun, Katrina R. Hamilton, Cecilia L. Bergeria, Andrew S. Huhn, Traci J. Speed, Ryan Vandrey, Kelly E. Dunn

**Affiliations:** 1 Department of Psychiatry and Behavioral Sciences, Johns Hopkins University School of Medicine, Baltimore, MD USA; 2 Arizona State University, Edson College of Nursing and Health Innovation, Phoenix, AZ USA; 3 Behavioral Pharmacology Research Unit, Department of Psychiatry and Behavioral Sciences, Johns Hopkins University School of Medicine, Baltimore, MD USA

**Keywords:** Translational research, Pharmacology

## Abstract

The potential synergistic effects of combining cannabinoids and opioids for analgesia has received considerable attention. No studies to date have evaluated this combination in patients with chronic pain. The present study aimed to evaluate the combined analgesic and drug effects of oral opioid (hydromorphone) and delta-9-tetrahydrocannabinol (dronabinol), as well as their effects on physical and cognitive functioning, and human abuse potential (HAP) outcomes among individuals with knee osteoarthritis (KOA). This was a within-subject, double-blind, randomized, placebo-controlled study. Participants (*N* = 37; 65% women; mean age = 62) diagnosed with knee osteoarthritis of ≥3/10 average pain intensity were included. Participants received (1) placebo-placebo, (2) hydromorphone (4 mg)-placebo; (3) dronabinol (10 mg)-placebo, and (4) hydromorphone (4 mg)-dronabinol (10 mg). Clinical and experimentally-induced pain, physical and cognitive function, subjective drug effects, HAP, adverse events, and pharmacokinetics were evaluated. No significant analgesic effects were observed for clinical pain severity or physical functioning across all drug conditions. Little enhancement of hydromorphone analgesia by dronabinol was observed on evoked pain indices. While subjective drug effects and some HAP ratings were increased in the combined drug condition, these were not significantly increased over the dronabinol alone condition. No serious adverse events were reported; hydromorphone produced more mild adverse events than placebo, but hydromorphone + dronabinol produced more moderate adverse events than both placebo and hydromorphone alone. Only hydromorphone impaired cognitive performance. Consistent with laboratory studies on healthy adults, the present study shows minimal benefit of combining dronabinol (10 mg) and hydromorphone (4 mg) for analgesia and improving physical functioning in adults with KOA.

## Introduction

The potential synergistic effects of combining cannabinoids and opioids for analgesia have drawn significant attention following preclinical evidence that cannabinoids and opioid combinations have additive nociceptive benefit with limited adverse effects [1], compared with larger doses of each drug alone [2]. A meta-analysis of 19 animal studies reported opioids combined with delta-9-tetrahydrocannabinol (THC) produced analgesic effects at opioid doses 9.5 lower than analgesia following greater doses of opioids administered alone [3].

Evidence for the cannabinoid-opioid synergistic effects in human laboratory studies remains tenuous, however [4, 5]. Most human laboratory studies have enrolled healthy adults and found limited opioid analgesic effects and/or no significant enhancement of analgesia following THC/opioid co-administration [6–8]. Although some studies, including our previous Phase II clinical trial with healthy adults, have reported limited THC enhancement of opioid effects, enhancement was only observed in low THC dose conditions (e.g., dronabinol 2.5 mg), and the cannabinoid-opioid combinations also increased human abuse potential (HAP) ratings, dysphoric effects, and adverse events, suggesting limited clinical utility for analgesia [9, 10]. A limitation of existing studies is their focus on healthy adults rather than persons with chronic pain, thus limiting our nascent understanding of the potential benefits of cannabinoid-opioid combinations in clinical populations. Moreover, a recent meta-analysis revealed significant variability in analgesic efficacy of cannabinoids in laboratory with healthy adults vs. observational studies with adults with chronic pain [11]. Finally, despite accumulating evidence that sex can moderate the efficacy of cannabinoid-based analgesia [12], prior studies did not examine potential sex (assigned at birth) differences.

The present study rigorously evaluated the combined effects of *dronabinol* (oral synthetic THC suspended in sesame oil), a partial agonist at the cannabinoid 1 and 2 receptors, and *hydromorphone*, a prototypic opioid agonist at the mu-opioid receptor, among individuals with knee osteoarthritis (KOA). KOA is an ideal chronic pain condition in which to examine the combined effects of cannabinoid and opioid in a laboratory study for a number of reasons. KOA is a leading cause of chronic pain and disability worldwide, and prevalence is expected to sharply rise due to the increasing population age and rate of obesity [13]. Patients with KOA have substantial clinical pain and poor physical functioning, and commonly used treatments (e.g., non-steroidal anti-inflammatory drugs, opioids) have limited benefits and long-term risks [14].

Using a within-subject, double-blinded, randomized, and placebo-controlled design, the present study examined the independent and combined effects of dronabinol and hydromorphone on experimentally-induced acute and chronic pain models, clinical pain, physical and cognitive functioning, HAP ratings, and adverse events. A subset of participants provided blood samples that were analyzed for pharmacokinetic profiles, and the potential moderating effects of sex across outcomes were explored. To our knowledge, this is the first behavioral pharmacology study to examine the drug effect profile and analgesic response, as well as physical and cognitive functioning following co-administration of oral THC with an opioid in a clinical chronic pain sample.

## Materials and methods

### Participants

**Fig. 1:**
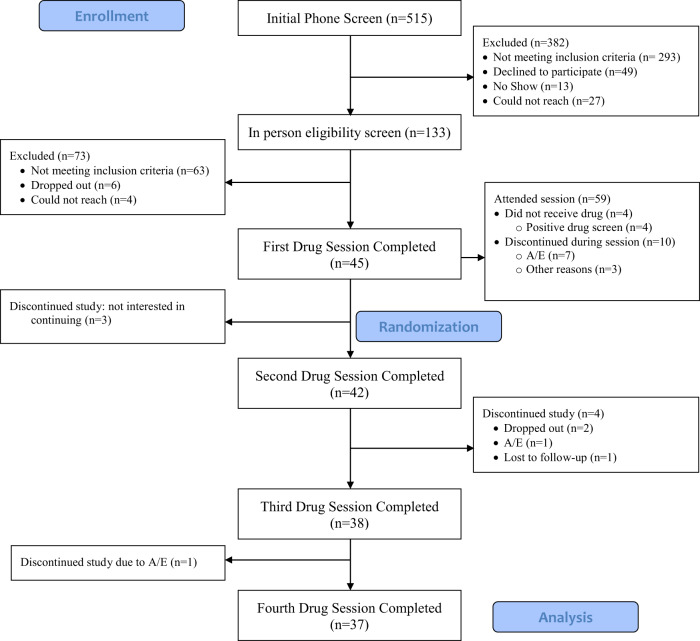
Study consort diagram. A/E adverse event.

Individuals with KOA were recruited between 11/2017 and 12/2020 using locally posted and online flyers and print/radio advertisements. A CONSORT flow diagram is depicted in Fig. 1. The protocol was approved by the Johns Hopkins School of Medicine IRB and registered with clinicaltrials.gov (NCT03098563) and all participants provided informed consent to participate.

### Study design and procedure

This Phase II study used a randomized, double-blind, placebo-controlled, within-subject study design. Potential participants completed a phone and in-person screening, during which they provided a urine sample that was tested for recent drug exposure and pregnancy (for premenopausal females) and were excluded if they reported past month opioid exposure. Eligibility was determined based on medical history and physical including ECG, hepatic, hematologic, and chemistry functioning (see Online Supplement Table S1). A knee x-ray was conducted to determine Kellgren–Lawrence score [15] which is a five-point ordinal scale (from none [0] to severe [4]) grading schema for classifying the severity of KOA. Participants also completed self-report measures and were introduced to quantitative sensory testing (QST) during screening. Neither participants nor staff were informed about the specific medications under investigation (a list of six medications were provided as possible study drugs during the informed consent process).

### Experimental study sessions

Eligible participants completed four experimental study sessions scheduled ≥7 days apart. All sessions started around 0800. Participants were asked to refrain from over-the-counter medications on session days and maintain steady doses of prescribed and non-contraindicated medications throughout participation. After providing a urine sample testing negative for drugs and pregnancy (as applicable), participants received a calorie and fat-controlled (~10 g) breakfast. Participants (*n* = 11) who opted into pharmacokinetic sampling received an intravenous line in the non-dominant arm. Around 0930, participants completed baseline QST, function (physical and cognitive), and self-report measures lasting ~60 min. Study drugs (e.g., two oral capsules) were co-administered around 1100, and QST, function, and self-report measures were repeated at 60, 120, 180, and 240 min post dosing. Medications were administered at the same time (versus based upon timing of peak effects) to reflect likely clinical practice.

### Measures

#### QST methods

A number of sensory pain measures were included in this study to quantify acute pain (i.e., threshold responses, temporal summation of pain, cold pressor testing, and conditioned pain modulation), and as a model of chronic pain (i.e., capsaicin [10% topical cream]).

#### Acute pain measures

All thermal and pressure pain testing was conducted over two trials to obtain consistent readings; trial results were averaged for each measure. **Thermal pain**: To assess thermal threshold and tolerance, thermal stimulation via thermode (Medoc TSA II, Israel) was applied gradually from a pre-set baseline (31 °C) at a rate of. 5 °C/s (i.e., ascending method of limits paradigm). To identify thermal threshold, participants were asked to indicate when the task “first feels painful” and press a button to terminate the task. Thermal tolerance was assessed over two identical trials where the participant indicated that the perceived pain was intolerable. **Pressure pain**: A pressure algometer (Somedic; Sweden) delivered steady, constant pressure to the upper trapezius muscle until the maximum limit of 1200 kPa was reached or the participant indicated pain. **Temporal summation**: Repetitive thermal and punctuate stimuli were delivered to assess temporal summation. To assess thermal temporal summation, the thermode delivered 10 heat pulses (0.5-s each) at 49 °C and 51 °C at inter-pulse intervals of 2.5 s. After each pulse, participants rated the sensation on a 0–100 (‘no sensation’ to ‘intolerable pain’) scale. Thermal temporal summation is reported as the average of the initial pain rating minus the maximum pain rating for each temperature. Mechanical temporal summation was determined by applying weighted pinprick stimulators with fixed stimulus intensities to a flat contact area of 0.2 mm diameter on the ventral forearm. Participants verbally indicated their pain (0–100) following a single stimulus with a force of 256 mN or 512 mN. Thereafter, participants indicated their peak pain over a 10-stimulus train that lasted 10-s. Mechanical temporal summation scores were calculated by taking the average of two wind-up ratios that were determined by adding 1 (to avoid dividing by 0) to each rating for both probe weights and dividing the peak pain reported following the train of ten stimuli by the initial rating from a single stimulus. **Cold pressor tasks**: Participants were asked to rate their pain every 30-s while their hand was submerged in a circulating cold-water bath (5 °C). Both cold pain threshold (time to first pain) and tolerance (time to hand withdrawal) were also assessed. The cold pressor was then repeated for 20 s followed by concurrent randomly ordered pressure pain or mechanical temporal summation tasks in order to assess conditioned pain modulation. According to task, initial pressure pain threshold/peak mechanical temporal summation pain were subtracted from those values obtained during the combined cold pressor task. **After sensation**: Lingering pain 15-s following thermal and mechanical temporal summation and cold pressor was assessed.

#### Chronic pain measure

As reported previously [16], 10% topical capsaicin cream coupled with thermal stimuli was used to model chronic pain. **Baseline sensitization period**: An open square raised adhesive frame was placed around the capsaicin site before the cream was distributed on the skin to allow for 30-min absorption. Once the capsaicin cream was removed, 45 °C thermal stimulation was applied to the affected site for 5 min coupled with pain ratings (0–100) every minute. Then, the affected area was assessed for flare, secondary hyperalgesia, and mechanical temporal summation. **Rekindling**: At each QST assessment, the thermode at 45 °C heated the treatment site for 5 min, thereafter, flare, secondary hyperalgesia, thermal threshold and mechanical temporal summation were reassessed.

#### Global QST outcomes

Two global QST outcomes were derived from the full testing battery: (1) Central Sensitization (average Z-scores of thermal and mechanical temporal summations, conditioned pain modulation, and after-sensation ratings) and (2) General Sensitivity (average Z-scores of pressure and thermal thresholds, thermal tolerance, cold pressor threshold and tolerance), with higher values representing greater sensitization and sensitivity, respectively.

#### Clinical pain severity

Clinical pain severity rating was collected at every time point using an online (delivered via Qualtrics) 0–100 Visual Analog Scale (VAS) [17] which included a straight line with one end indicating “no pain at all” and the other end indicating “the most intense pain imaginable.”

#### Self-reported drug effects

Following FDA guidance [18], participant drug effect ratings (i.e., Drug Effect, Good Effect, Bad Effect, High, Like the Way I Feel, and Nausea) were collected via 0–100 VAS.

#### HAP measures

The primary HAP outcome was whether participants achieved (yes/no) a post-drug exposure rating of ≥60 on the 0-100 VAS High scale [18]. Participants also rated whether they enjoyed study medications (“yes,” “no,” and “no effect”), the dollar amount they would pay for the medication, and the likelihood they would take the medication again on a 6-point Likert scale (0 = “not at all” to 5 = “extremely”).

#### Physical function measures

Three measures of objective physical performance [19] were administered: (1) 2-min walking distance task [20], (2) Timed Up and Go test (time to rise from a standard chair, walk 3 m, and return to a seated position) [20], and (3) stair climb total (time to ascend and descend two-step stairs with hand rails) [19].

#### Cognitive function measures

Three tasks assessed cognitive functioning [21–23]: (1) Psychomotor Ability (percent correct on the Digit Symbol Substitution Task [DSST], where participants used a keypad to replicate patterns displayed on a computer screen); (2) Working Memory (mean reaction time and percent correct on the Paced Serial Addition Task [PASAT] where participants added sequentially presented integers together in rapid sequence); (3) Fine Motor Movement (maximum number correct on a circular light test where participants repeated visual patterns displayed on a board over 60-s).

#### Adverse events (AEs)

Participants were asked whether they experienced any side effects of the study medication throughout the session. Reported AEs were documented and classified according to severity (i.e., mild, moderate, and severe) and relatedness (i.e., unrelated, possibly, probably, and definitely). Primary outcomes were the total number of related AEs, collapsed across severity, as well as the number of related AEs rated as mild, moderate, or severe.

#### Pharmacokinetic analyses

Whole blood samples were collected in standard vacutainer tubes free of additives for participants (*n* = 11) who participated in this optional procedure consisting of baseline (pre-drug) and 10 post-drug collections at 30-min intervals up to 5 h. Immediately following collection, samples were aliquoted into storage tubes and frozen at –80 °C until analysis. Liquid chromatography and tandem mass spectrometry (LC–MS/MS) was used to determine maximum concentration (*C*_max_) and time to maximum concentration (*T*_max_) for THC, hydromorphone, and metabolites, as a function of study condition.

### Study medications

Oral hydromorphone (4 mg, Sky Pharma), dronabinol (10 mg; Akorn), and placebo were overencapsulated using size 00 gelcaps to blind drug condition to participants and experimenters. Hydromorphone was chosen as a prototypical opioid with limited differential CYP450 metabolism [24]. The doses for hydromorphone and dronabinol were selected because they were within the range approved by the FDA for clinical use (and thus could be prescribed clinically) and were hypothesized to yield analgesic effects [9]. The study included one control condition: placebo + placebo (i.e., placebo), and three experimental conditions: (1) hydromorphone + placebo (i.e., hydromorphone); (2) dronabinol + placebo (i.e., dronabinol); and (3) hydromorphone + dronabinol. The first session was fixed to the hydromorphone condition to ensure participants safely tolerated hydromorphone before receiving the combination with dronabinol. Session order thereafter was randomized by a research pharmacist who had no study-related interactions, using a random sequence generator.

### Power analysis

A power analysis was derived from a prior evaluation of oxycodone and smoked cannabis on cold pressor tests [10]. With power = 0.8, alpha = 0.05 and expected effect size (Cohen’s *d* = 1.15), the power analysis determined a sample size of 15 would be sufficient to detect large effects of drug condition. A sample of *N* = 30 was planned to support analyses of sex differences.

### Data analytic plan

Primary outcomes (i.e., peak or trough ratings post-drug administration) as a function of drug were examined with mixed-effects models for continuous outcomes, generalized estimating equations (GEE) for dichotomous outcomes, and multinomial logistic regression for nominal categorical outcomes (i.e., response option for study medication enjoyment was “yes,” “no,” and “no effect”). Chi-square analysis was conducted when GEE models did not converge. Drug conditions were compared using Tukey post-hoc tests. Analyses were replicated using area under the curve (AUC) analysis and including body mass index (BMI) as a covariate. Findings did not significantly change so only primary findings with peak effect analysis without controlling for BMI are reported. Sex did not moderate any study outcomes, so only main effects are reported. Main data analyses were conducted by an independent biostatistician who did not participate in outcome assessments. For all analysis SAS version 9.4 was used and alpha was set at 0.05, two tailed.

## Results

### Participant characteristics

As shown in Table 1, participants (*N* = 37; *M*_age_ = 61.8 ± 6.7) were predominantly female, White or Black, and not of Hispanic origin.

**Table 1:** Participant characteristics at baseline.

Total *N*	37
Age, mean (*SD*)	61.8 (6.7)
Sex assigned at birth, *n* (%)	
Male	13 (35.1)
Female	24 (64.9)
Race, *n* (%)	
White	19 (51.4)
Black	15 (40.5)
Asian	0 (0)
American Indian/Alaska Native	0 (0)
Native Hawaiian/Pacific Islander	0 (0)
Multiracial	1 (2.7)
Prefer not to answer	2 (5.4)
Hispanic ethnicity, *n* (%)	0 (0)
Relationship status, *n* (%)	
Never married	9 (24.3)
Married or remarried	14 (37.8)
Divorced, separated, or widowed	14 (37.8)
Education level, *n* (%)	
Less than high school	6 (16.2)
High school graduate	7 (18.9)
Some college, no degree	12 (32.4)
College or professional degree	10 (27.0)
Above college	5 (13.5)
Yearly household income, *n* (%)	
$0–$24,999	9 (24.3)
$25,000–$49,999	12 (32.4)
$50,00–$74,999	7 (18.9)
$75,000–$99,999	4 (10.8)
$100,000 or more	2 (5.4)
Prefer not to answer	3 (8.1)
Disability, *n* (%)	8 (21.6)
Currently receiving pain treatment, *n* (%)	9 (24.3)
Lifetime history of prescription opioid use, *n* (%)	23 (62.2)
Body Mass Index, mean (*SD*)	34.1 (6.3)
Average pain severity (0–10 scale from BPI), mean (*SD*)	5.2 (2.1)

*BPI* Brief Pain Inventory.

### QST outcomes

#### Acute pain outcomes

**Fig. 2:**
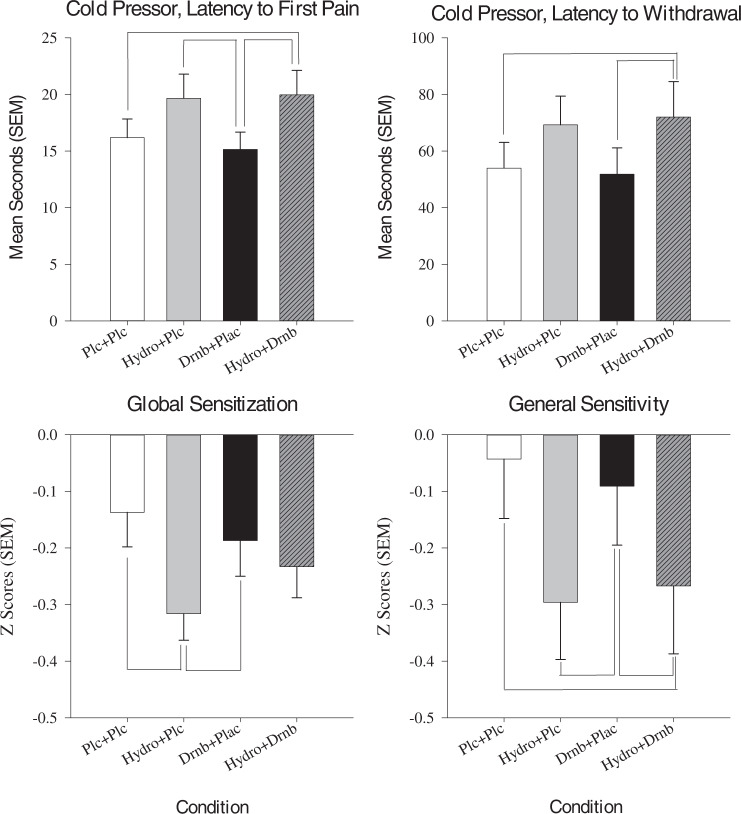
Quantitative sensory testing (QST) outcomes. Data show results from the cold pressor task (top) and global QST measures (bottom), as a function of study condition (x-axis). Medication conditions were Placebo + Placebo (Plc + Plc), oral hydromorphone 4 mg + placebo (Hydro + Plc), and hydromorphone 4 mg combined with oral dronabinol 10 mg (Hydro + Drnb). Line that connect bars represents a significant difference in post-hoc comparison and error bars represent SEM.

There was limited evidence of dronabinol enhancement of hydromorphone on QST (see Table 2, Fig. 2). A significant main effect of drug was found on pressure pain threshold (*p* = 0.018) whereby hydromorphone showed greater analgesia than placebo (*p* = 0.009). No drug-related differences in thermal threshold and tolerance, and mechanical or thermal temporal summation (*p*’s > 0.05) were found. A significant main effect of drug on cold pressor threshold (*p* = 0.001) was observed, such that hydromorphone + dronabinol increased cold pressor threshold more than placebo (*p* = 0.038) and dronabinol (*p* = 0.007), but not more than hydromorphone (*p* = 0.986). Hydromorphone also significantly increased cold pressor threshold compared to dronabinol (*p* = 0.018). Similar results were found for cold pressor tolerance (*p* = 0.002), such that hydromorphone + dronabinol significantly increased tolerance more than placebo (*p* = 0.018) and dronabinol (*p* = 0.011), but not hydromorphone (*p* = 0.946). None of the drug conditions significantly altered conditioned pain modulation (*p*s > 0.05).

**Table 2:** Summary of primary outcomes.

Primary outcomes	Placebo		Hydromorphone 4 mg, oral		Dronabinol 10 mg, oral		Hydromorphone 4 mg, oral + Dronabinol 10 mg, oral		*P* value (partial eta^2^)
	Mean/%	SEM	Mean/%	SEM	Mean/%	SEM	Mean/%	SEM	
Quantitative sensory testing									
Acute pain model									
Pressure pain threshold (0–1200 kPa)	446.27^a^	27.10	535.32^a^	30.52	488.05	34.05	492.18	34.16	**0.018 (0.048)**
Heat pain threshold (**°**C)	44.63	0.48	45.24	0.37	44.85	0.50	45.13	0.49	0.216 (0.010)
Heat pain tolerance (**°**C)	48.06	0.27	48.15	0.23	48.02	0.24	48.27	0.27	0.453 (−0.002)
Mechanical temporal summation	3.90	0.63	3.44	0.55	5.23	1.17	5.10	1.58	0.322 (0.004)
Thermal temporal summation	0.59	0.09	0.66	0.10	0.84	0.23	0.75	0.11	0.503 (0.004)
Cold pressor threshold (time in s)	16.19^a^	1.65	19.66^b^	2.14	15.16^b,c^	1.52	19.97^a,c^	2.16	**0.001 (0.085)**
Cold pressor threshold severity rating (0–100 VAS)	59.44	5.75	57.98	5.15	64.91	4.93	60.54	5.06	0.153 (0.017)
Cold pressor tolerance (time in s)	53.99^a^	9.08	69.27	10.15	51.84^b^	9.32	72.04^a,b^	12.51	**0.002 (0.080)**
Conditioned pain modulation, mechanical temporal summation	−9.89	2.91	10.73	2.99	−14.41	3.63	−15.43	3.33	0.437 (−0.002)
Conditioned pain modulation, pressure pain threshold	100.51	15.94	120.14	15.39	109.30	15.10	98.84	8.96	0.781 (−0.013)
Chronic pain model									
Capsaicin, thermal threshold	39.55	0.47	39.36	0.46	38.84^a^	0.39	40.25^a^	0.44	**0.001 (0.058)**
Capsaicin, mechanical temporal summation	1.83	0.31	1.51	0.10	1.66	0.22	1.75	0.18	0.700 (−0.011)
Global QST measures									
Central sensitization (z-score)	−0.14^a^	0.06	−0.32^a,b^	0.05	−0.19^b^	0.06	−0.23	0.05	**0.004 (0.072)**
General pain sensitivity (z-score)	−0.04^a,b^	0.10	−0.30^a,c^	0.10	−0.09^c,d^	0.10	−0.27^b,d^	0.12	**<0.001 (0.102)**
Clinical pain severity (0–100 VAS)	12.57	2.92	9.32	2.71	13.46	2.93	10.95	2.74	0.302 (0.005)
Physical functioning tests									
2-min walking distance	289.46	11.41	295.97	12.62	283.09	11.22	285.84	13.95	0.058 (0.031)
Tug time	11.90	0.67	12.20	0.71	12.00	0.71	11.91	0.62	0.620 (−0.008)
Stair time	6.18	0.52	6.13	0.48	5.98	0.49	6.04	0.43	0.861 (−0.015)
Participant ratings (0–100 VAS)									
Drug Effect	16.35^a,b^	3.70	26.06^c,d^	4.61	52.92^a,c^	5.80	57.57^b,d^	5.32	**<0.001 (0.338)**
Good Effect	23.38^a^	4.94	40.60^a^	5.85	39.59	5.67	34.70	5.14	**0.018 (0.048)**
Bad Effect	5.92^a,b^	2.16	7.54^c,d^	2.48	33.16^a,c^	5.82	38.35^b,d^	6.26	**<0.001 (0.270)**
High	7.03^a,b^	2.52	15.03^c,d^	4.01	40.62^a,c^	5.93	37.24^b,d^	5.19	**<0.001 (0.264)**
Like the Way I Feel	49.59	5.37	59.86	5.01	52.03	5.47	45.14	5.73	0.137 (0.018)
Nausea	1.54^a^	0.65	7.14	3.13	7.32	2.92	15.65^a^	4.21	**0.012 (0.055)**
Human abuse potential (HAP) measures									
Enjoyed medication (% Yes)	21.2^a,b^		35.5		40.6^a^		38.5^b^	0.11	**0.001 (0.328)**
Would take medication again (0–5)	0.79	0.19	1.39	0.24	1.09	0.26	1.19	0.29	0.185 (0.013)
≥60 on “High” rating scale (%)	0%^a,b^		11.8%^c,d^		47.1%^a,c^		47.1%^b,d^		**0.002 (0.389)**
Willingness to pay for medication ($)	17.34	9.74	16.29	7.34	18.50	8.83	24.31	13.49	0.827 (-0.014)
Cognitive testing									
Circular lights (max per minutes)	33.11^a^	1.55	29.50^a^	1.54	32.33	1.85	32.16	1.60	**0.029 (0.042)**
DSST (proportion correct)	0.60	0.06	0.58	0.07	0.68	0.05	0.64	0.07	0.491 (-0.004)
PASAT, mean reaction time correct (sec)	1694.87	38.00	1748.40	63.84	1731.30	29.72	1788.52	34.80	0.343 (0.003)
PASAT, correct (%)	43.67^a^	4.07	34.98^a^	4.30	40.57	3.78	42.19	4.05	**0.023 (0.045)**

Outcomes represent mean peak ratings or percent participants for each condition (*N* = 37). Matching superscripts indicate significant (*p* < 0.05) differences in post-hoc comparisons. Partial eta^2^ effect sizes provided for significant results: small (0.01), medium (0.06), large (0.14).

*SEM* standard error of the mean, *DSST* Digit Symbol Substitution Task, *PASAT* Paced Auditory Serial Addiction Task, *VAS* Visual Analog Scale.

Statistically significant *p*-values are in bold.

#### Chronic pain outcomes

There was a significant main effect of drug on heat pain threshold in the zone of primary hyperalgesia sensitized by capsaicin (*p* = 0.001). Hydromorphone + dronabinol significantly increased heat pain threshold in this area more than dronabinol (*p* = 0.005), but not placebo (*p* = 0.253) or hydromorphone (*p* = 0.140). There were no drug condition differences for mechanical temporal summation on the sensitized area (*p* = 0.700).

#### Global QST outcomes

A significant main effect of drug was revealed on Central Sensitization (*p* = 0.004). Hydromorphone significantly reduced central sensitization compared to placebo (*p* = 0.002) and dronabinol (*p* = 0.043), but not hydromorphone + dronabinol (*p* = 0.317). There was also a significant drug condition main effect on Global Sensitivity ratings (*p* < 0.001), where hydromorphone significantly reduced general pain sensitivity relative to placebo (*p* = 0.003) and dronabinol (*p* = 0.016). Hydromorphone + dronabinol also significantly reduced general pain sensitivity relative to placebo (*p* = 0.010) and dronabinol (*p* = 0.044), but not hydromorphone (*p* = 0.981).

### Clinical pain severity outcome

Baseline (prior to drug administration) clinical pain severity ratings for each drug condition were: (1) 22.51/100 (placebo); (2) 31.54/100 (hydromorphone); (3) 26.17/100 (dronabinol); and (4) 26.57/100 (hydromorphone + dronabinol). Paired *t*-tests revealed that there were no statistically significant differences in baseline clinical pain severity ratings across drug conditions (*p* values ranging from 0.06 to 0.95). No significant drug condition main effect on peak clinical pain severity (*p* = 0.302) was observed.

### Physical functioning outcomes

No significant drug condition main effects on 2-min walking distance, tug time, or stair time (*p* > 0.05) were observed.

### Self-reported drug effect outcomes

**Fig. 3:**
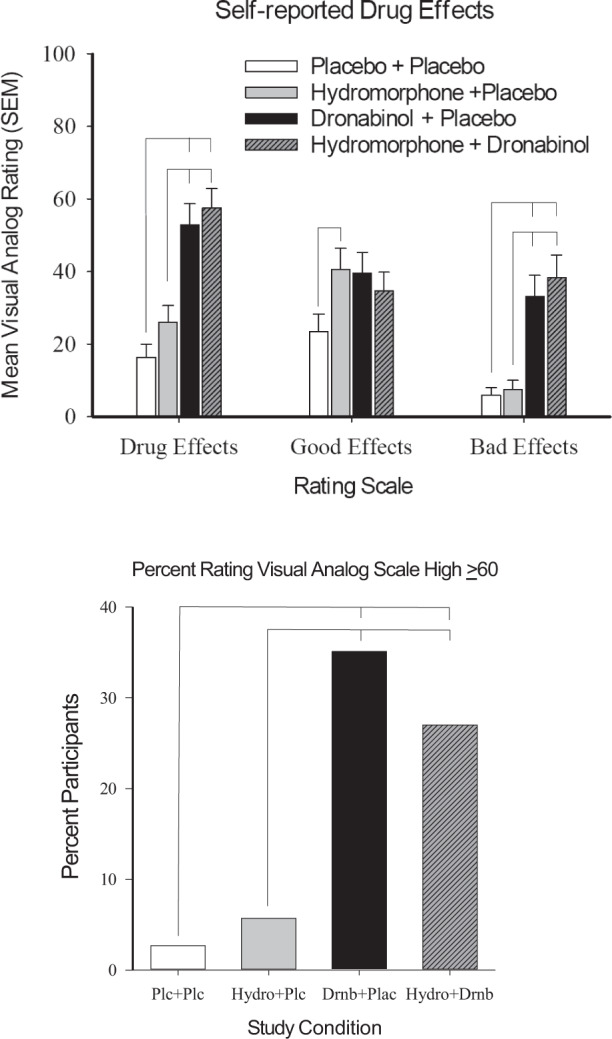
Participant ratings. Data show results from participant ratings of Drug, Good, and Bad Effects and percent of participants rating their feeling of High on a VAS 60 or higher during a session, as a function of study condition. Line that connect bars represents a significant difference in post-hoc comparison and error bars represent SEM.

Overall, drug conditions produced subjective ratings that were significantly different from placebo (Table 2 and Fig. 3). All active drug conditions except hydromorphone significantly increased ratings of Drug Effect over placebo (*p*’s < 0.001), though hydromorphone + dronabinol was not elevated relative to dronabinol (*p* = 0.646). Ratings for Good Effect (*p* = 0.018 for main effect) were significantly higher than placebo for hydromorphone (*p* = 0.019), but hydromorphone did not differ significantly from dronabinol (*p* = 0.052) or hydromorphone + dronabinol (*p* = 0.227). Bad Effect ratings (*p* < 0.001 for main effect) were significantly higher than placebo for dronabinol and hydromorphone + dronabinol (*p* < 0.001) conditions; Bad Effect ratings of dronabinol and hydromorphone + dronabinol were also higher than hydromorphone (*p* < 0.001). Nausea ratings (*p* = 0.012 for main effect) were significantly higher for hydromorphone + dronabinol than placebo (*p* < 0.005), but hydromorphone and dronabinol did not differ from placebo (*p* > 0.05). No significant differences were observed for Like the Way I Feel (*p* = 0.137). Both dronabinol and hydromorphone + dronabinol, but not hydromorphone alone, significantly increased ratings of High when compared with placebo (*p* < 0.001); hydromorphone + dronabinol did not increase High relative to dronabinol (*p* = 0.929).

### HAP outcomes

A significant main effect of drug condition was observed for enjoyment of study medications (*p* = 0.001). Participants were more likely to report enjoying study medication in the dronabinol (*p* = 0.003) and the hydromorphone + dronabinol (*p* = 0.003) conditions than not having any drug effects. A significant main effect of drug condition on the percent of participants rating High ≥60 (*p* < 0.001) was also found in the dronabinol and hydromorphone + dronabinol conditions, relative to placebo and hydromorphone (Fig. 3). There was no significant drug condition effect on interest in taking medication again or amount of money participants were willing to pay for the drug (*p* > 0.05).

### Cognitive function

A significant drug condition difference was observed on the circular lights task (*p* = 0.029) where hydromorphone significantly decreased accuracy relative to placebo (*p* = 0.043). Dronabinol and hydromorphone + dronabinol did not significantly deviate from placebo (*p* > 0.05). No drug condition differences were observed for psychomotor ability (*p* > 0.05). However, a significant main effect was observed for working memory (*p* = 0.023), such that hydromorphone significantly decreased working memory relative to placebo (*p* = 0.025); dronabinol and hydromorphone + dronabinol did not significantly deviate from placebo (*p* > 0.05).

### Adverse events (AEs)

Study-related AEs were documented in 52 (35.1%) sessions and experienced by 26 (70.3%) participants. No serious AEs occurred. Compared with placebo, active drug dosing produced more mild and moderate AEs (*p* < 0.05). Specifically, a higher proportion of participants experienced mild AEs due to hydromorphone (*p* = 0.006) compared to placebo. In the case of moderate AEs, hydromorphone+dronabinol was more likely to produce moderate AEs as compared to placebo (*p* = 0.028) and hydromorphone (*p* = 0.011). The online supplement Table S2 provides the frequency of AEs (separated by the severity) for each drug condition.

### Pharmacokinetics (PK)

Compared to each drug alone, hydromorphone + dronabinol did not significantly impact maximum, or time to maximum THC or hydromorphone concentrations (*p* > 0.05). However, both Tmax for the metabolites 11-hydroxy-Δ9-tetrahydrocannabinol and hydromorphone-3-β-d-glucuronide trended towards significance (*p* = 0.05 and *p* = 0.06, respectively), with the hydromorphone + dronabinol achieving maximum concentration more quickly than other conditions. See Tables S3 and S4 in the online Supplementary Data.

## Discussion

The present study rigorously and comprehensively evaluated the effects of co-administering dronabinol (10 mg) and hydromorphone (4 mg) on evoked and clinical pain, self-reported drug effects, HAP metrics, physical and cognitive functioning, and AEs in patients with chronic pain. Overall, our findings reveal limited clinical benefits, and suggest that co-administering dronabinol (10 mg) with hydromorphone (4 mg) to this clinical population demonstrated a slightly enhanced risk for use along with elevated risk for AEs in the combined condition. These findings were consistent across sexes (see online supplement Table S5 for each outcome’s peak mean and standard deviation separated by sex).

We found that although hydromorphone + dronabinol was associated with significant analgesia on several QST outcomes, it was not different than hydromorphone by itself, indicating no added benefit of the combination at the doses tested. We also found that none of the drug conditions significantly changed ratings of clinical pain severity. Overall, these findings are consistent with previous laboratory studies with healthy adults demonstrating limited additive benefit of combined cannabinoids and opioids on analgesia [6–8, 11]. However, there remain significant differences in findings observed in human vs. preclinical/animal models. Findings from preclinical models show overall robust enhancements of opioids effects by a wide-range of cannabinoids [3]. The reason for these discrepancies is vastly unknown, but may be attributable to differences in the cannabinoids being administered in preclinical versus human studies. For instance, none of the human studies employed weight-based cannabinoid dosing [6–10]. In addition, as noted in our recent systematic review on analgesic effects of cannabis/cannabinoids, some additional factors such as, different cannabinoid compounds, routes of administration, chronicity of dosing, and cannabis use history may contribute to the variability in cannabinoids’ analgesic effects [25].

To our knowledge, the present study is the first to evaluate the individual and combined effects of dronabinol and hydromorphone on clinically-relevant standardized physical testing measures [26, 27]. None of the drug conditions improved or diminished these outcomes. This finding may be related to the null effects observed for clinical pain severity, as knee pain can directly impact performance on these physical functioning tests [26]. Nevertheless, these data undermine support for using dronabinol and/or hydromorphone for improving physical functioning in patients with KOA at the tested doses. However, it is uncertain whether these null results can be generalized to other chronic pain conditions, or to persons for whom these drugs do reduce clinical pain severity.

Relative to hydromorphone, dronabinol significantly increased the percent of participants who rated feeling High ≥ 60, a cutoff discussed by HAP experts as a metric of future risk. This value is higher than what was observed in prior studies [9] and provides initial evidence that this dose could engender use in the tested population. However, this concern is tempered by the lack of effect on other HAP ratings, including interest in taking medications again, assigned dollar value, or ratings of “Like the Way I Feel”, combined with elevated Bad Effects and Nausea ratings. The negative ratings produced by these medications are also reflected in the AE trends, wherein hydromorphone + dronabinol produced the highest rate of moderate AEs. Interestingly, only the hydromorphone condition decremented cognitive performance, and these results replicate our prior findings with healthy adults [9]. Ultimately, these data mirror prior studies that found no overarching benefit, and slightly enhanced risk of side effects or future use when dronabinol was combined with hydromorphone [6–8]. Longer-term follow-up with those who co-use these drugs may shed further light on understanding their relative HAP and side effects of co-use.

Despite several strengths, the present study had a number of limitations. First, only a single dose of hydromorphone and dronabinol was used, precluding dose-dependent examinations. A more parametric, dose-dependent design was considered but rejected due to concerns about feasibility of completing numerous sessions given that participants were older adults with chronic pain. In lieu of that design, the study assessed the highest cannabinoid dose tested by our prior research to provide maximal opportunity to detect the presence of any effect (whereas it would be more challenging to infer whether lack of effect smaller doses represented true lack of effect or a dose-related issue). The more definitive test for this line of research would be a full dose-dependent evaluation. In addition, the average participant age was >60 years old, which has been associated with changes in drug metabolism that may have impacted results. Third, use of an oral synthetic THC formulation may limit the data generalizability. Many advocates for the synergistic properties of cannabis prefer different routes of administration (e.g., inhalation) arguing that the whole, natural cannabis flower produces different effects than THC alone and could interact with opioids or produce analgesia in ways we do not yet fully understand. Fourth, although necessary for safety purposes, the hydromorphone condition was unrandomized which may have introduced order effects. Fifth, a longer walking test (6-min vs. 2-min) may have increased clinical pain levels and revealed more nuances in physical performance, but was shortened due to participant burden. Lastly, we focused on the acute effects of dronabinol and hydromorphone co-administration, and thus we cannot inform the long-term use of these two drugs on clinical outcomes, side effects, and HAP.

## Conclusion

To our knowledge, this is the only laboratory study to examine the independent and combined effects of doses of dronabinol (10 mg) and hydromorphone (4 mg) that are within the range that can be prescribed therapeutically on a comprehensive array of clinically-relevant outcomes in individuals with chronic pain. These data extend our prior research with healthy adults by providing further evidence that combining dronabinol with hydromorphone for analgesia and physical function yields little benefit in a clinical cohort. The doses tested for dronabinol did not improve hydromorphone’s analgesic profile but did increase ratings of High, as well as negative side effects, and AEs. Finally, any significant analgesic effect observed following dronabinol + hydromorphone appeared to be driven by the hydromorphone, negating dronabinol enhancement of analgesia at the dose tested.

### Supplementary information

Supplemental Material
