# Supplementary material for: Within-subject, double-blind, randomized, placebo-controlled evaluation of combining the cannabinoid dronabinol and the opioid hydromorphone in adults with chronic pain
Source: Neuropsychopharmacology. 2023 May 18;48(11):1630–8. doi: 10.1038/s41386-023-01597-1 (PMC10516978; doi:10.1038/s41386-023-01597-1)
Supplement: Supplementary file 1 — Supplemental Material [file 41386_2023_1597_MOESM1_ESM.docx]

**Table S1. Study Eligibility Criteria**

| **Inclusion Criteria:** |
| --- |
| - Aged 45+ - Met American College of Rheumatology KOA criteria - Report average pain level >3/10 - No opioid medications during the past 30 days - Urine sample tests negative for common illicit substances (e.g., amphetamine, barbiturates, benzodiazepines, buprenorphine, cannabis, cocaine, methadone, methamphetamine, MDMA, opioids, phencyclidine, tramadol) and pregnancy - Medically cleared to take study medications - Willing to comply with the study protocol |
| **Exclusion Criteria:** |
| - Cognitive impairment preventing study completion - Meet DSM-5 criteria for alcohol/substance use disorder - Meet criteria for lifetime history of opioid use disorder - Recent or significant history of cannabis use - Have taken gabapentinoid, tricyclic antidepressants venlafaxine, duloxetine, stimulants, or benzodiazepines - Self-report any illicit drug use in the past 7 days - Presence of any clinically significant medical/psychiatric illness judged by the investigators to put subject at elevated risk for experiencing an adverse event, including suicidal ideation - Current peripheral neuropathy, Raynaud’s phenomenon, vasculitis or severe peripheral vascular disease, infection, periodic limb movement disorder or restless leg syndrome, systemic inflammatory or autoimmune disorders (e.g., rheumatoid arthritis, lupus), seizure disorder, or a chronic pain condition that produces pain greater than their KOA pain - Have a known allergy to the study medications or sesame seed oil - Pregnant or breast feeding - Taking medications contraindicated with hydromorphone or dronabinol - Have a history of clinically significant cardiac arrhythmias or vasospastic disease |
| **Trail Stopping Rules^*^:** |
| - Systolic blood pressure: >180mm Hg - Diastolic blood pressure: >120mm Hg - Heart Rate: > submaximal heart rate (220 – [age x 0.85)]. - Not abiding by study policies and procedures - Development of intercurrent illness or condition that altered participant risk profile |
| *Participants were evaluated to determine continuation of the study if any of these issues arose |

**Table S2. Frequency of Adverse Events (AEs) Across Drug Conditions**

| **Severity of AE** | **Condition** | **Count/%** |
| --- | --- | --- |
| Mild AE | Placebo + Placebo | 2 (5.4%) |
|  | Hydromorphone + Placebo | 12 (32.4%) |
|  | Dronabinol + Placebo | 8 (21.6%) |
|  | Hydromorphone + Dronabinol | 8 (21.6%) |
| Moderate AE | Placebo + Placebo | 1 (2.7%) |
|  | Hydromorphone + Placebo | 1 (2.7%) |
|  | Dronabinol + Placebo | 5 (13.5%) |
|  | Hydromorphone + Dronabinol | 9 (24.3%) |
| Severe AE | Placebo + Placebo | 0 (0%) |
|  | Hydromorphone + Placebo | 0 (0%) |
|  | Dronabinol + Placebo | 0 (0%) |
|  | Hydromorphone + Dronabinol | 0 (0%) |

**Table S3**. **Maximum concentration (C_max_) for study drugs and metabolites in each study condition**

|  | **THC-COOH** | **THC** | **11-OH THC** | **HYM** | **HYM-GLUC** |
| --- | --- | --- | --- | --- | --- |
| **Hydromorphone** | 0 | 0 | 0 | 2.80 | 15 |
| **Dronabinol** | 23 | 1.78 | 1.89 | 0 | 0 |
| **Combination** | 15.09 | 0.82 | 1.46 | 2.46 | 18.27 |

*Note*. THC-COOH = 11-Nor-9-carboxy-Δ^9^-tetrahydrocannabinol the main secondary metabolite of tetrahydrocannabinol; THC = tetrahydrocannabinol; 11-OH THC = 11-Hydroxy-Δ^9^-tetrahydrocannabinol the main active metabolite of tetrahydrocannabinol; HYM= hydromorphone; HYM-GLUC = hydromorphone-3-β-d-glucuronide metabolite of hydromorphone.

**Table S4. Time to maximum concentration (T_max_) in hours for study drugs and metabolites in each study condition**

|  | **THC-COOH** | **THC** | **11-OH THC** | **HYM** | **HYM-GLUC** |
| --- | --- | --- | --- | --- | --- |
| **Hydromorphone** | 0 | 0 | 0 | 1.30 | 2.40 |
| **Dronabinol** | 2.89 | 1.44 | 2.11 | 0 | 0 |
| **Combination** | 2.86 | 0.77 | 1.05 | 1.09 | 1.68 |

*Note*. THC-COOH = 11-Nor-9-carboxy-Δ^9^-tetrahydrocannabinol the main secondary metabolite of tetrahydrocannabinol; THC = tetrahydrocannabinol; 11-OH THC = 11-Hydroxy-Δ^9^-tetrahydrocannabinol the main active metabolite of tetrahydrocannabinol; HYM= hydromorphone; HYM-GLUC = hydromorphone-3-β-d-glucuronide metabolite of hydromorphone.

**Table S5. Peak Effect Means and Standard Deviations for Study Outcomes Across Sexes**

| Primary Outcomes | Placebo | | Hydromorphone 4mg, oral | | Dronabinol 10mg, oral | | Hydromorphone 4mg, oral+Dronabinol 10mg, oral | |
| --- | --- | --- | --- | --- | --- | --- | --- | --- |
|  | Mean/%  Female | Mean/%  Male | Mean/%  Female | Mean/%  Male | Mean/%  Female | Mean/%  Male | Mean/%  Female | Mean/%  Male |
| Quantitative Sensory Testing |  |  |  |  |  |  |  |  |
| *Acute Pain Model* |  |  |  |  |  |  |  |  |
| Pressure Pain Threshold  (PPTh; 0-1200) | 412.4 (168.8) | 508.7 (142.8) | 492.6 (183.9) | 614.2 (167.9) | 430.0 (180.3) | 595.3 (217.3) | 431.0 (168.5) | 605.1 (232) |
| Heat Pain Threshold  (HPTh) | 44.9 (2.9) | 44.1 (2.9) | 45.1 (2.6) | 45.6 (1.4) | 45.0 (2.7) | 44.6 (3.7) | 45.3 (2.8) | 44.8 (3.4) |
| Heat Pain Tolerance | 48.1 (1.2) | 47.9 (2.3) | 48.0 (1.2) | 48.4 (1.7) | 47.9 (1.2) | 48.2 (1.9) | 48.3 (1.4) | 48.2 (2.2) |
| Mechanical Temporal  Summation (MTS) | 4.0 (4.4) | 3.7 (2.6) | 3.2 (3.5) | 3.9 (3.3) | 5.1 (6.3) | 5.6 (8.7) | 5.9 (11.8) | 3.6 (2.2) |
| Thermal Temporal  Summation | 0.6 (0.6) | 0.6 (0.6) | 0.7 (0.6) | 0.7 (0.6) | 0.7 (0.6) | 1.1 (2.3) | 0.8 (0.6) | 0.6 (0.7) |
| Cold Pressor Threshold  (time in seconds) | 16.0 (10.5) | 16.5 (9.5) | 19.6 (13.1) | 19.9 (13.3) | 15.3 (9) | 14.9 (10.1) | 20.6 (14.6) | 18.8 (10.2) |
| Cold Pressor Threshold  Severity Rating (0-100) | 57.6 (26.1) | 63.2 (22.4) | 49.6 (26.3) | 69.7 (19.1) | 61.4 (19.4) | 70.9 (25) | 54.8 (22.6) | 69.8 (22.3) |
| Cold Pressor Tolerance  (time in seconds) | 56.4 (63.3) | 49.6 (38.0) | 74.3 (70.6) | 60 (41.7) | 57.2 (66.0) | 42.0 (33.4) | 81.2 (87.2) | 55.2 (48.3) |
| Conditioned Pain  Modulation with MTS | -5.5 (9.1) | -3.8 (5.9) | -7.3 (13.3) | -9.2 (15.5) | -12.5 (23.6) | -2.2 (2.3) | -9.0 (15.0) | -4.1 (5.2) |
| Conditioned Pain  Modulation with PPTh | 84.9 (82.6) | 129.4 (117.2) | 97.6 (79.0) | 161.7 (106.9) | 90 (67.9) | 144.8 (119.9) | 96.3 (58.6) | 103.5 (48.0) |
| *Chronic Pain Model* |  |  |  |  |  |  |  |  |
| Capsaicin, HPTh | 40.2 (2.8) | 38.4 (2.6) | 39.9 (2.7) | 38.3 (2.6) | 39.0 (2.3) | 38.6 (2.6) | 40.6 (2.6) | 39.5 (2.9) |
| Capsaicin, MTS | 1.5 (0.5) | 2.4 (2.8) | 1.5 (0.7) | 1.5 (0.5) | 1.4 (0.4) | 2.1 (2) | 1.8 (1.3) | 1.7 (0.8) |
| *Global QST Measures* |  |  |  |  |  |  |  |  |
| Central Sensitization  (z-score) | -0.1 (0.4) | -0.1 (0.4) | -0.4 (0.2) | -0.2 (0.4) | -0.2 (0.3) | -0.1 (0.4) | -0.2 (0.2) | -0.2 (0.3) |
| General Pain Sensitivity  (z-score) | -0.1 (0.6) | 0 (0.6) | -0.3 (0.7) | -0.3 (0.4) | 0 (0.6) | -0.2 (0.8) | -0.3 (0.8) | -0.3 (0.7) |
| Clinical Pain Severity  (0-100 VAS) | 10.6 (16.9) | 16.2 (19.4) | 6.1 (13.1) | 15.3 (20.7) | 9.6 (15) | 20.6 (20.9) | 8.2 (14.2) | 16.0 (20.1) |
| Physical Functioning Tests |  |  |  |  |  |  |  |  |
| 2-min walking distance | 269.2 (55.3) | 326.9 (79.1) | 276.5 (58) | 330.4 (92.4) | 265.5 (53.1) | 311.5 (75.1) | 258.2 (60.3) | 329.7 (84.2) |
| Tug time | 12.6 (3.9) | 10.6 (4.2) | 12.5 (3.6) | 11.6 (5.4) | 12.5 (4.4) | 11.2 (3.7) | 12.7 (3.4) | 10.6 (3.2) |
| Stair time | 6.8 (3.5) | 5.0 (2.1) | 6.6 (2.9) | 5.2 (2.7) | 6.5 (3.2) | 5.1 (2.1) | 6.7 (2.6) | 5.0 (1.8) |
| Participant Ratings  (0-100 VAS) |  |  |  |  |  |  |  |  |
| Drug Effect | 13.3 (23.2) | 21.9 (20.9) | 24.4 (27.1) | 29.6 (28.6) | 54.0 (38.0) | 50.8 (31) | 60.1 (34) | 52.8 (29.9) |
| Good Effect | 21.8 (32.0) | 21.9 (20.9) | 42 (35.3) | 37.5 (34.7) | 36.8 (37.1) | 44.8 (29.6) | 35.5 (31.2) | 33.2 (32.6) |
| Bad Effect | 5.0 (14.3) | 7.5 (11.1) | 9.0 (16.6) | 4.3 (9.1) | 35.7 (36.7) | 28.5 (33.8) | 47.4 (40.0) | 21.7 (28.9) |
| High | 3.6 (9) | 13.4 (22) | 13.3 (24.2) | 18.8 (23.4) | 40.5 (39.5) | 40.8 (30.3) | 34.1 (33.4) | 43.1 (28.2) |
| Like the Way I Feel | 48.0 (36.4) | 52.5 (25.3) | 59.2 (32.2) | 61.3 (24.3) | 51.8 (36.2) | 52.5 (28.5) | 46.1 (36.2) | 43.3 (33.6) |
| Nausea | 1.2 (2.9) | 2.1 (5.5) | 8.8 (21.9) | 3.5 (6.3) | 6.5 (19.2) | 8.9 (15.3) | 21.5 (29.3) | 4.8 (11) |
| Human Abuse Potential (HAP) Measures |  |  |  |  |  |  |  |  |
| Enjoyed medication (%  Yes) | 13.0% | 40.0% | 30.4% | 37.5% | 30.4% | 66.7% | 31.6% | 57.1% |
| Would take medication  again | 0.8 (1.2) | 0.8 (1.0) | 1.4 (1.3) | 1.2 (1.6) | 1 (1.4) | 1.4 (1.6) | 1.2 (1.6) | 1.3 (1.3) |
| >60 on "High" rating scale  (%) | 0% | 7.7% | 4.2% | 9.1% | 37.5% | 30.8% | 25.0% | 30.8% |
| Willingness to pay for  medication ($) | 22.0 (64.6) | 5.6 (6.8) | 20.2 (44.3) | 4.4 (4.4) | 24.1 (58.3) | 4.2 (4.7) | 28 (80) | 14.3 (18.8) |
| Cognitive Testing |  |  |  |  |  |  |  |  |
| Circular lights  (max per minutes) | 33.7 (10.9) | 32.1 (6.2) | 29.3 (10) | 29.9 (8.1) | 31.8 (12.6) | 33.2 (8) | 31.4 (10.8) | 33.4 (5.4) |
| DSST (proportion correct) | 0.6 (0.4) | 0.6 (0.4) | 0.7 (0.4) | 0.4 (0.3) | 0.6 (0.3) | 0.8 (0.3) | 0.7 (0.4) | 0.6 (0.4) |
| PASAT, mean reaction time  correct (sec) | 1678.1 (253.2) | 1727 (115) | 1784.2 (185.5) | 1663.4 (555.4) | 1716.3 (185.3) | 1758.6 (125.1) | 1758.6 (217.4) | 1842.4 (86.3) |
| PASAT, correct (%) | 41.5 (27.7) | 48.1 (10.6) | 33.7 (25.3) | 38.3 (17.5) | 39.6 (24.5) | 42.5 (15.4) | 40.4 (26.7) | 45.9 (9.3) |

*Note*. DSST = Digit Symbol Substitution Task, PASAT = Paced Auditory Serial Addiction Task, VAS = Visual Analogue Scale.
